# Comparative Study of Domoic Acid and Okadaic Acid Induced - Chromosomal Abnormalities in the CACO-2 Cell Line

**DOI:** 10.3390/ijerph2006030001

**Published:** 2006-03-31

**Authors:** Pinto-Silva Carvalho, R. Catia, Serge Moukha, William G. Matias, Edmond E. Creppy

**Affiliations:** 1 Department of Environmental Engineering - Toxicological Laboratory of the Federal University of Santa Catarina, Brazil Campus Universitário, Trindade, 88040-900, Brazil; 2 Unit of Mycology and Food Safety, National Institute of the Agronomic Research - INRA - Villenave d’Ornon, France.; 3 Department of Pharmaceutical Sciences - Toxicological Laboratory of the University of Bordeaux 2, France.

**Keywords:** Okadaic acid, Domoic acid, Micronuclei, Clastogenicity, Aneugenicity

## Abstract

Okadaic Acid (OA) the major diarrheic shellfish poisoning (DSP) toxin is known as a tumor promoter and seems likely implicated in the genesis of digestive cancer. Little is known regarding genotoxicity and carcinogenicity of Domoic Acid (DA), the major Amnesic Shellfish Poisoning (ASP) toxin. Both OA and DA occur in seafood and are of human health concerns. Micronuclei (MN) arise from abnormalities in nuclear division during mitosis due to a failure of the mitotic spindle or by complex chromosomal configurations that pose problems during anaphase. In order to evaluate the ability of okadaic acid (OA) and domoic acid (DA) to induce DNA damage we performed the micronucleus assay using the Caco-2 cell line. To discriminate between a clastogenic or aneugenic effect of OA and DA, the micronucleus assay was conducted by cytokinesis-block micronucleus assay using cytochalasin B with Giemsa staining and/or acridine orange staining, in parallel to fluorescence *in situ* hybridization (FISH) using a concentrated human pan-centromeric chromosome paint probe. Our results showed that OA and DA significantly increased the frequency of MN in Caco-2 cells. The MN caused by OA are found in mononucleated cells and binucleated cells, whereas those caused by DA are mainly in binucleated cells. The results of FISH analysis showed that OA induced centromere-positive micronuclei and DA increased the percentage of MN without a centromeric signal. In conclusion, both OA and DA bear mutagenic potential as revealed in Caco-2 cells by induction of MN formation. Moreover, OA induced whole chromosome loss suggesting a specific aneugenic potential, whereas DA seems simply clastogenic. At present, one cannot rule out possible DNA damage of intestinal cells if concentrations studied are reached *in vivo*, since this may happen with concentrations of toxins just below regulatory limits in case of frequent consumption of contaminated shell fishes.

## Introduction

Among marine phytoplankton about 40 species have the capacity of producing potent toxins [1]. Among these toxins, okadaic acid (OA) and domoic acid (DA) are considered important since they accumulate in filter-feeding animals consumed by humans, therefore representing an environmental and public health problem [2, 3]. Both could be ingested either separately or in combination with different types of sea foods. The cytotoxic, neurotoxic and genotoxic effects of OA have been verified by some authors [4–7] whereas Jeffery et al (2004) observed that only very few data on the possible genotoxicity and toxicology of DA are available, [8].

Intestinal tissue is the main target of OA in mammals, where it induces desquamation and diarrhea [4]. Before it is distributed to the brain, DA crosses the intestinal barrier, where it may cause some gastro intestinal disorders also. Thus, intestinal cells seem to be suitable to use to study both phycotoxins in vitro. Caco-2 cells from that point of view have been intensively used to study several toxicants that are normally ingested for their transport, biotransformation and mode of action in vitro [9–11].

OA is known as a tumor promoter [2, 3] and seems likely implicated in digestive cancer, [12]. Little is known regarding the genotoxicity and carcinogenicity of Domoic acid [8].

Micronuclei (MN) are nuclear abnormalities which have been used as an indicator of chromosome damage for over 20 years. MN arises from fragments that fail, because of the lack of a centromere, to incorporate into daughter nuclei during cell division [13]. MN can also be formed by entire chromosomes that lag behind during mitosis due to a failure of the mitotic spindle or by complex chromosomal configurations that pose problems during anaphase [14]. Thus, a micronucleus will contain either a chromosomal fragment or a whole chromosome. The cytokinesis – block micronucleus (CBMN) assay using Cytochalasin B in combination with the fluorescence in situ Hybridation (FISH) technique (with mitomycin C as positive control) with centromeric probes, has been developed to characterize the origin of MN occurring either spontaneously, or following exposure to various chemical agents, [15, 16].

The MN assay and FISH using chromosome-specific centromeric probes have met widespread acceptance both as screening tools for genotoxic compounds and for monitoring of humans exposure to environmental carcinogens, [17, 18]. The usefulness of the MN and FISH assay can be further enhanced by the incorporation of cytotoxicity measures, such as counts for apoptotic and necrotic cells [17], and by the detection of centromeres in micronucleus to differentiate between aneugens causing whole chromosome loss and clastogens which produce acentric fragments, [15, 18].

The present experiments were designed to evaluate genotoxic effects of DA and to discriminate between clastogenic and/or aneugenic effect of both Okadaic acid and Domoic acid using the CBMN with cytochalasin B assay combined with FISH technique on a human intestinal cell line Caco-2, using a range of concentrations that are cytotoxic to these cells as determined by MTT test.

## Materials and Methods

### Cell Culture

Caco-2 cells, a human colorectal adenocarcinoma cell line, were obtained from Dr. Jing Yu, Tufts School of Medicine (Medford, Mass., USA). The cells were grown as monolayer cultures in a humidified atmosphere of 95% air/5% CO_2_ in high glucose (25 mM) DMEM supplemented with 8 mM glutamine, penicillin (100 IU/ml), streptomycin (100 μg/ml), and 10% heat-inactivated fetal calf serum at 37°C.

### Chemicals

Okadaic acid (OA), domoic acid (DA) and Mytomicin C (MMC), Cytochalasin B were purchased from Sigma Chemical Company (St Quentin Fallavier, France). OA was dissolved in ethanol and DA was dissolved in MilliQ Water. Concentrated Human Pan-Centromeric Chromosome Probe was purchased from Cambio (UK).

### Cell Treatment

Exponentially growing cells were plated in a 6-well plate on glass coverslips (1.5 × 10^4^ cells/cm^2^) and cultured for 24 h prior to toxins treatments. Duplicate coverslips were established for each concentration. Cells were exposed to OA and DA for 24 hours, at concentrations of 15, 30, 60 and 100ng/ml.Cytochalasin was added at a concentration of 5 μg/ml (10 nM) together with the tested substances. The control cultures received an equivalent amount of the solvent. Mytomicin C (MMC) has been used as positive control (0.5μg/ml).

### MTT Assay

Cell viability was determined using the MTT assay. In viable cells, MTT is converted to the purple formazan dye, which is measured spectrophotometrically following dissolution; No conversion occurs in dead cells. The Cells were plated in 96-well microplates, following 72 h contact with OA (15, 30, 60 and 100ng/ml). At the end of the treatment, the medium was replaced by 20 μl medium containing 0.5 mg/ml MTT and the plates were kept for 3 h in the incubator. After a PBS wash, the formazan was solubilized with 1N HCl–isopropanol (1/23; v/v) and the absorbance was read at 540 nm using a Microplate Autoreader (MR4000, Dynatech, St. Cloud, France). Cell viability was expressed as a percentage of the control.

### Micronucleus Assay

Cells were cultured at 37° C for 24 h. At the end of the incubation the cultures were centrifuged (250×*g*, 10 min) resuspended in buffer (0.9 mM NH_4_HCO_3_ and 132 mM NH_4_Cl) for 20 min at room temperature and centrifuged for 15 min at 250×*g*. This procedure was repeated twice. Cells were then fixed in cold fixative (methanol: acetic acid, 3:1) for 20 min at room temperature. Samples for microscopic observation were obtained by carefully dropping the cell suspension onto clean wet slides. Microscopic slides were stained with either Giemsa or acridine orange (Sigma, France) or hybridized within 1 week of preparation.

### Fluorescence in Situ Hybridization (FISH)

Centromeric FISH was performed using a concentrated human Pan-centromeric chromosome probe for all centromeres (Star FISH – Cambio –UK). The slides were allowed to age for at least 3 days and treated with 10% pepsine (Sigma) in 10 mM HCl for 10 min at 37°C. The slides were then washed briefly in distilled water and phosphate-buffered-saline (PBS) and postfixed for 10 min at room temperature with 3% formaldehyde (Sigma, France) in PBS. They were then washed with PBS and dehydrated in an 80%–90%–95% ethanol series. DNA denaturation was performed in 70% formamide (Sigma) in 2xSSC (saline–sodium citrate buffer) at 70°C for 2 min and dehydrated in the same ethanol series of increasing concentration.

The hybridization mixture containing the probe (2.5μg/ml) and 500μg/ml salmon sperm DNA (Salmon testes DNA, Sigma) in 2 x SSC, was denatured at 80°C for 5 min, followed by chilling on ice for 4 min. An aliquot of 12.5μl per slide was applied, which were then covered with coverslips and sealed with rubber cement.

Hybridization was performed for 16 h at 37°C in a moist chamber. After the incubation, the slides were washed two times in 2xSSC for 4 min, and then in Tween-20 (Sigma) buffer for 5 min. The slides were then incubated with the blocking reagent (5% skimmed milk in 4xSSC) at 37°C for 10 min. The slides were washed with 4× SSC, covered with a 1:250-dilution of anti-biotin antibody (Sigma) in IB (immunological buffer: 0.5% skimmed milk in 4xSSC) and incubated at 37°C for 30 min. After a wash in Tween-20 buffer, the slides were incubated in a 1:20-dilution of FITC-conjugated anti-mouse antibody (Sigma), followed by incubation with a 1:20-dilution of FITC-conjugated anti-sheep antibody (Sigma) for 30 min at 37°C.

All incubations were performed in a moist chamber, and were followed by washes in the Tween-20 buffer. After the last wash the slides were dehydrated with the increasing series of ethanol and stained with propidium iodide (5μg/ml, Sigma).

### Microscopic Analysis

Acridine orange or Giemsa-stained slides were coded and scored blind under a magnification of 400x. A total of 1000 cells with preserved cytoplasm were scored for each concentration. The MN frequency was calculated as the number of micronucleated cells.

For FISH analyses the slides were scored with a fluorescence microscope (Olympus BX40) attached to a video camera equipped with a UV filter block and connected to a personal computer-based image analysis system. The micronuclei present in the cells were examined for the presence of one or more centromeric signals and were classified as centromere-positive (CEN+) or centromere-negative (CEN−). A total of 100 MN were scored for each concentration.

### Statistical Analysis

The data of MN are expressed as means±SD for at least three independent determinations in triplicate for each experimental point. The data from controls and exposed groups (OA and DA) were compared by non-parametric two-tailed Kruskal-Wallis test. For FISH analysis, differences between means and between the percentages of CEN+ and CEN− were evaluated by the non-parametric Mann–Whitney test. A *P* value of less than 0.05 was considered as statistically significant.

## Results

OA clearly decreases Caco-2 cells viability as measured by MTT assay with an IC_50_ of 15 ng/ml as previously reported [9]. Similarly, DA decreased cell viability with a higher IC_50_ of about 70 ng/ml). Concentrations in this range have then been used for further experiments.

**Figure 1:**
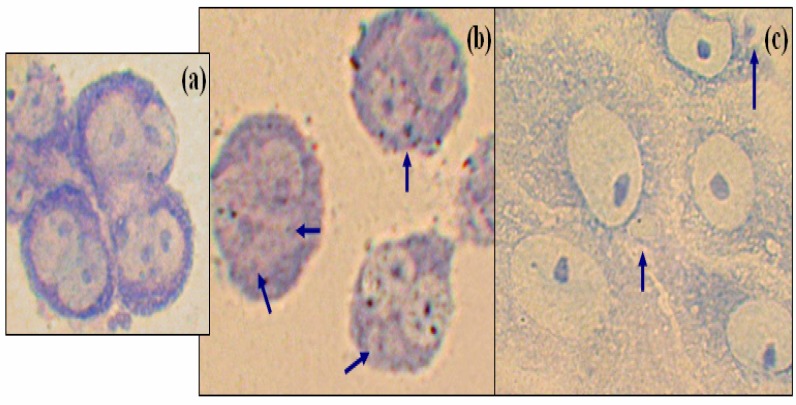
(a) Control cells 24h following the addition of Cytochalasin B showing binucleated cells, (400X), (b) MN in binucleated cells treated by Domoic acid, (100 ng/ml); (c) MN in mononucleated cells treated by okadaic acid, (60 ng/ml). Cells are stained with Giemsa (arrows indicate micronuclei)

The cytokinesis-block micronucleus assay (CBMN) has been performed with cytochalasin B, which prevents cytokinesis, resulting in polynucleated cells. The number of nuclei per cell indicates the number of nuclear divisions that have occurred since the addition of cytochalasin B. Binucleated cells could be observed in the control cultures, (Fig. 1a). MN are observed in DA-treated binucleated cells that have finished one nuclear division, (Fig. 1b). The numbering of these among 1000 cells revealed that cells treated with DA showed 56% of MN in binucleated cells, (Fig 1b). Concerning okadaic acid, only few binucleated cells bear MN (less than 1%) as compared to the control with cytochalasin B alone (0.35%) cells (results not shown). Up to 50 and 58% for high concentrations show MN in mononucleated cells, (Fig. 1c). Figure 1a, b and c show examples of MN in different conditions of treatment, experimental values are shown in table 1.

**Figure 2:**
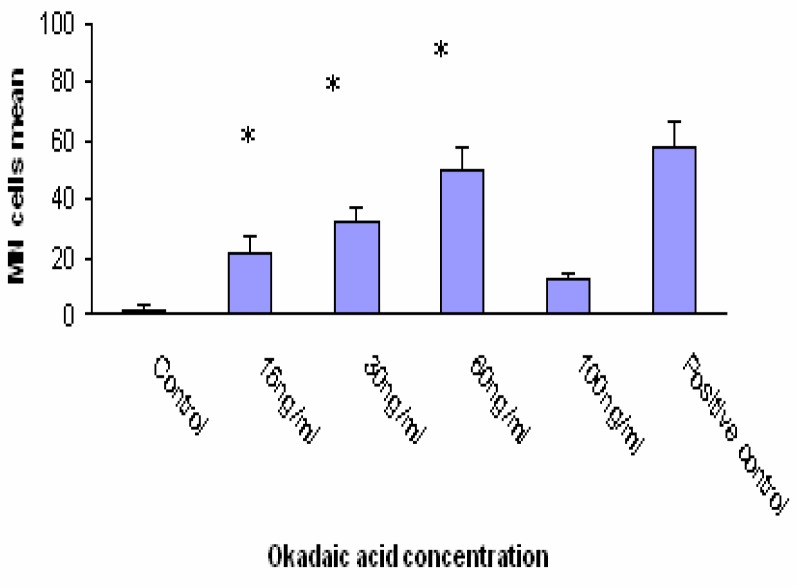
MN frequency in Caco-2 cells exposed to OA. Data are expressed as mean ± SD. *indicates significant differences as compared to negative control (*P*<0.05).

**Figure 3:**
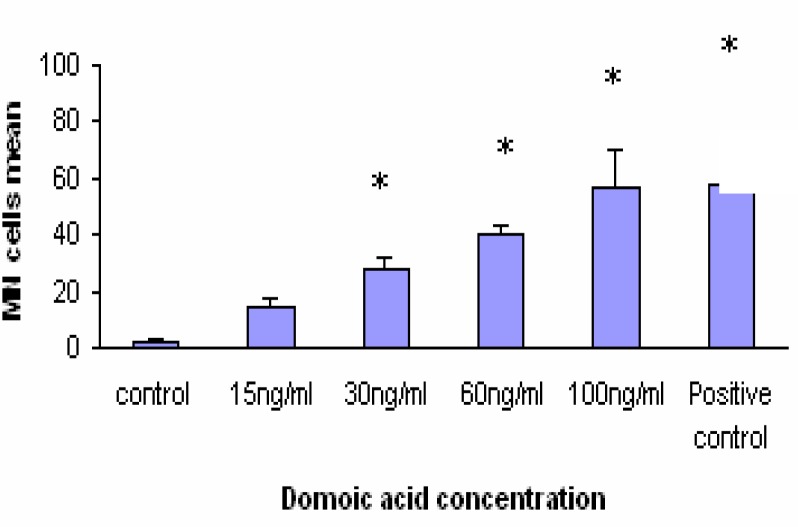
Micronuclei (MN) frequency in Caco-2 cells exposed to DA. Data are expressed as mean ± SD. *indicates significant differences as compared to negative control (*P*<0.05).

Significant differences in the incidence of MN were observed in Caco-2 cells exposed to 15, 30 and 60 ng/ml OA concentration. OA induced formation 50 % MN cells at concentration of 60ng/ml. The positive control (MMC 1.5 μM) induced the formation 58 % MN cells (Fig. 2). The results of MN assay after 24 h exposure to different DA concentrations are shown in Fig. 3. DA clearly caused a dose-dependant increase in MN frequency. At concentration of 100ng/ml DA induced formation 56% MN cells. The positive control (MMC 1.5 μM i.e. 500 ng/ml) induced the formation 58% MN cells. A comparison of MN formation rate in Caco-2 cells after 24 h incubation with different OA and DA concentrations shows similar shape for both toxins except at 100 ng/ml where OA-treated cells showed a marked decreased number of MN (compare Fig. 2 and Fig. 3).

**Figure 4:**
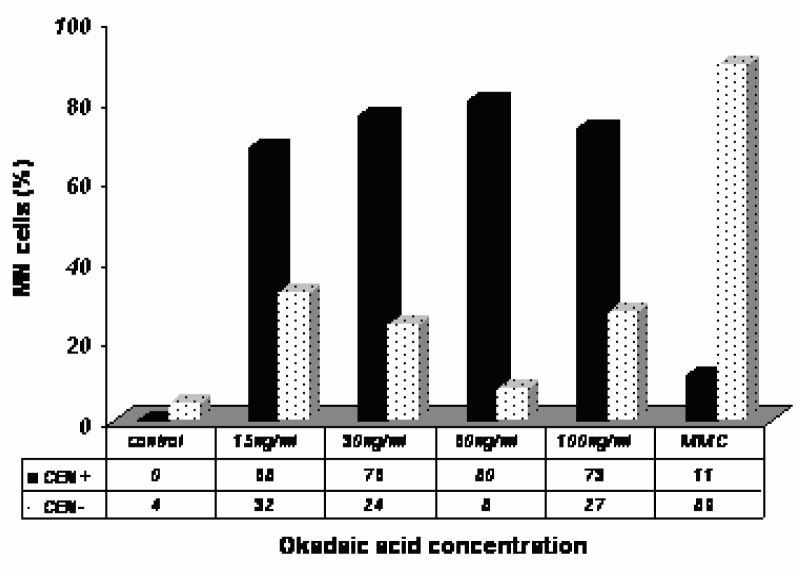
FISH analysis in Caco-2 cells after 24h exposure to OA. MN=number of micronucleated cells; CEN+= MN containing one or more centromeric signals (percentage); CEN−= MN containing no centromeric signal (percentage); MMC= Mytomicin C (clastogenic compound). Results presented are yielded by 3 independent experiments.

To determine the nature of the MN induced by OA and DA, we carried out a CBMN assay in combination with FISH using a centromeric DNA probe. The results obtained with the FISH analysis in Caco-2 cells exposed to OA are shown in Fig. 4. An increase in CEN+ in cells showing MN was found in OA-treated Caco-2 cells as compared to control, indicating a major contribution of CEN+ in the total MN scored in OA-treated cells.

**Figure 5:**
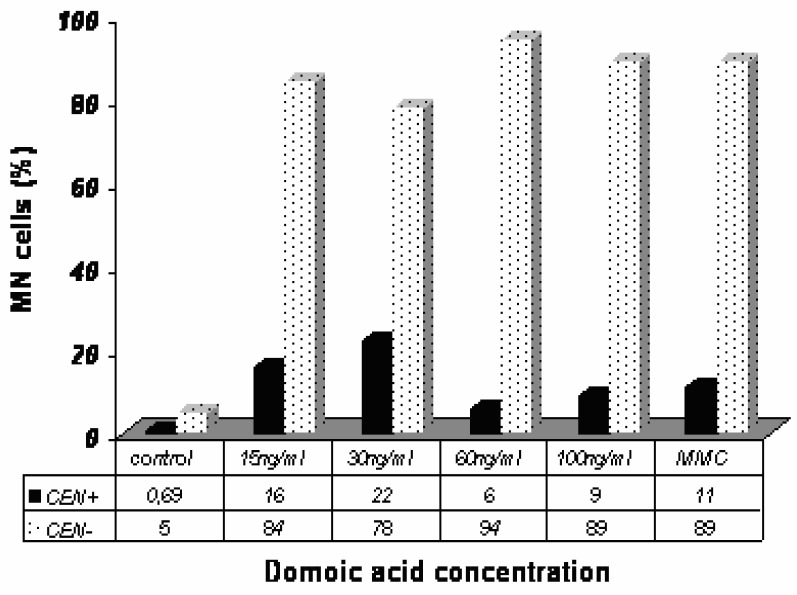
FISH analysis in Caco-2 cells after 24h exposure to DA. MN= number of micronucleated cells; CEN+= MN containing one or more centromeric signals (percentage); CEN−= MN without a centromeric signal (percentage); MMC= Mytomicin C (clastogenic compound). Results are means of 3 independent experiments.

The results obtained with the FISH analysis in Caco-2 cells exposed to DA are shown in Fig. 5. The frequency of CEN− increased in Caco-2 cells exposed to DA after 24 h.

The FISH technique permits to discriminate among the cells showing MN those having centromeric hybridization within OA or DA treated cells. On this criterion OA and DA show almost opposite images. The majority of MN in DA-treated cells proved to be centromer negative whereas the majority of MN in OA-treated cells is centromer positive.

Putting in parallel results of MN with cytochalasin B and Giemsa staining with results of FISH, OA seems rather to generate centromeric-labeled MN in mononucleated and/or binucleated cells whereas DA generates MN mainly in binucleated cells without centromeric labeling.

## Discussion

In the present study we confirmed the cytotoxicity of okadaic acid (OA) [9] and we showed that Domoic acid (DA) is also cytotoxic to the human intestinal cell line Caco-2. Using the respective cytotoxic concentrations we have evaluated the ability of these toxins to produce nuclear abnormalities in term of micronuclei (MN). First we observed MN in both mono and bi-nucleate cells using the cytokinesis-block micronuclei with Cytochalasin B and Giemsa staining. Next, to this technique we added the technique of *in situ* hybridization (FISH) with concentrated human pan-centromeric chromosome probe for the discrimination of MN arising from acentric fragments and from whole lagging chromosomes in Caco-2 cells exposed to okadaic acid and domoic acid.

The mechanistic approach combining CBMN with cytochalasin B and Giemsa staining and the FISH technique with Mitomycin C as positive control presents at least one advantage, in the coherence of the data obtained. Cytochalasin B indicates by the count of MN in mononucleated and binucleated cells the number of cell divisions that occurred during incubation with the toxins and thus discriminates aneugenicity and clostogenicity.

Our results showed that at OA concentrations of 15, 30 and 60 ng/ml the frequency of MN significantly increased in Caco-2 cells. However, the concentration of 100 ng/ml decreased the incidence of MN in cells to about 13%. This fact has to be attributed to OA cytotoxicity because this concentration induced apoptosis in Caco-2 cells as previously shown [19]. The reduction in viable cell number reflects apoptotic cell death. Traore et al. [19] and Rossini et al. [20] have proved that OA induces apoptosis in mammalian cells and specifically in human cancer cells [19–21] suggesting that the apoptosis induced by OA might be considered as a response of Caco-2 cells to this marine toxin, occurring at high concentrations. At concentrations of OA up to 15 ng/ml apoptosis is very rare.

Le Hegarat et al. [7] have observed that OA fails to induce direct DNA damage in vitro in CHO-K1 cells at the *Hprt* locus as well as primary DNA damage in rat hepatocytes. These authors demonstrated that the genotoxicity of OA triggers chromosome loss and/or non-disjunction and that its aneuploidic potential should be taken into account as a mode of action for its carcinogenic effect.

FISH analyses in Caco-2 cells exposed to OA showed that this toxin increases the percentage of MN containing one or more centromeric signals (CEN+). A centromeric signal in MN indicates that one entire chromosome resulting from chromosome mal-segregation (non-disjunction), has been lost [16]. Our results demonstrated that OA induced whole chromosome loss suggesting that OA is a compound with aneugenic potential. This result is in accordance with those obtained by Le Hegarat et al [7], Le Hegarat et al [22] suggested that aneuploidy occurs when replicated chromosomes fail to accurately segregate between the two daughter cells. The final result is the production of cells with abnormal number of chromosomes. In this context one may wonder what properties prevail in the present findings, genotoxic ones or epigenetic ones [23] or both. If only genotoxic properties are involved, one should find broken chromosomes. Instead MN are mostly found with the FISH technique as MN/CEN+. Aneugens could act on different cell targets, but disturbance of the mitotic spindle, kinetochores, centrosomes, microtubules and the anaphase promoting complex are mostly reported [14, 18].

Our results in DA-exposed cells showed a significant increase in MN frequency for 30, 60 and 100 ng/ml. This effect is a consequence of chromosomal aberrations and spindle disturbance, suggesting that DA is a genotoxic compound. The genotoxic response that DA caused in digestive glands cells has been demonstrated by Dizer et al. [24]. Conversely Rogers and Boyes [25] have previously reported that DA did not increase MN frequency in V79 Chinese hamster lung cells [25]. Our data do not permit any equivocation on the potential DNA damaging capacities of DA.

The FISH assay with pan-centromeric probes showed that DA increased the percentage of MN without a centromeric signal (CEN−) leading to a deficit in the percentage of centromere positive MN (CEN+) in Caco-2 cells exposed to DA. This was corroborated by the cytochalasin B-Giemsa staining assay which showed that DA induced predominantly MN in binucleated cells. These results suggest that DA induces clastogenic damage but is not aneugenic. No published studies have examined the in vivo mutagenicity and carcinogenicity of domoic acid [8].

The MN assay combined with FISH test revealed that, although OA and DA induce MN, OA is aneugenic, as is already known whereas DA is clastogenic. The consequences of these effects remain to be evaluated in vivo in experimental animal models. It appears that both Okadaic acid and Domoic acid bear mutagenic potential as revealed in Caco-2 cells by induction of MN formation.

Our data are in line with recent finding of Rosefort et al. [26] who showed that the aneugens diethylstilbestrol, griseofulvin, and vincristine sulphate, in very similar concentration range, increased MN frequencies in mononucleated and binucleated cells, whilst the clastogens mitomycin C (500 ng/ml), bleomycin, and doxorubicin, increased MN frequency only in binucleates [26]. Thus Domoic acid should rather be classified as a clastogenic agent.

MN formation in mononucleated is indicative of an aneugenic action. This was confirmed by centromere labelling using the FISH method. The results suggest that MN in mononucleates may be an interesting additional parameter to the CBMN assay. Future studies should clarify whether the micronucleated mononucleate cells have escaped the cytokinesis block and may become polyploid, as suggested by several authors, including Nesti et al. [15], Rosefort et al. [26], Surralles et al. [27], Ouanes et al. [28] and Ouanes et al. [29].

The FISH analysis showed that MMC induced mainly MN containing acentric fragments rather than whole chromosomes [15], in contrast to okadaic acid but similarly to domoic acid.

One may assume that consumers are exposed yearly to concentrations of DA and mainly OA corresponding to doses below the regulatory limits in sea foods that could be harmless (not diarrheic) if sufficiently low. There is nonetheless concern that residual levels of okadaic acid, a known tumour promoter that is the main DSP toxin present in coastal waters, might increase the risk of cancer among regular shellfish consumers. To test this hypothesis, Cordier et al. [12] conducted an ecological study linking digestive cancer mortality rates with a proxy measure of contamination by DSP toxins in 59 coastal areas in France at two time periods: 1984–1988 and 1989–1993. Using both Poisson regressions and test for trends of standardized mortality ratios across four exposure categories, they found some evidence of associations for several digestive cancer sites (oesophagus, stomach, colon, liver, and total digestive cancers for men; stomach and pancreatic cancers for women). Colon cancer rates among men remain statistically significant after eliminating possible confounding factors including alcohol [12].

In vitro assays show here that at 15 ng/ml OA and DA already induced either aneuploidism and/or micronuclei in progeny of Caco-2 cells that are from human intestinal tissues. Such a concentration can be reached in vivo when a person eats 500g of wet mussels containing 1.9 μg/g, i.e., lower than the regulatory levels (2 μg/g) if the toxins is linearly distributed in the body.

It is nevertheless true that OA is mainly distributed in the intestinal tissues and contents, [4] indicating that human intestine is the main site for possible cancer. The exposure to sub-regulatory concentrations are always possible that may not induce diarrhoea but obviously some effects such as aneuploidism and micronuclei could occur in intestinal tissues or elsewhere.

The tumour promoting ability of OA has been demonstrated for other sites such as skin in vivo with clear correlation with in vitro data, [2, 30].

At present, it could be postulated that the carcinogenic risk in humans has to be considered in relation to frequent consumption of mussels contaminated by these marine toxins, either alone or combined. The risk may be worsened by the presence of several heavy metals such as Cd, also contaminating mussels, that induces synergistic effects, [5].

Thus the risk linked to ingestion of DSP and ASP toxins, respectively potential tumor promoter and clastogen in human, has to be seriously considered or reconsidered by promoting future epidemiological studies.

## Figures and Tables

**Table 1:** Number of Micronuclei (MN) in mono and Binucleate Caco-2 cells (1000 cells counted) following incubation with both Domoic Acid (DA) and Okadaic Acid (OA)

*Toxin*	*Number of MN*	
	*Monucleated*	*Binucleated*
DA	580	560
OA	502	8
MMC	670	590
